# 1,3-Di-3-pyridyl-2,3-dihydro-1*H*-naphth[1,2-*e*][1,3]oxazine

**DOI:** 10.1107/S1600536808003887

**Published:** 2008-02-13

**Authors:** Betül Şen, Zuhal Turgut, Emel Pelit, Muhittin Aygün

**Affiliations:** a Department of Physics, Dokuz Eylül University, 35160 Buca-Izmir, Turkey; b Department of Chemistry, Yιldιz Technical University, 34210 Davutpaşa Campus-Istanbul, Turkey

## Abstract

In the crystal structure of the title compound, C_22_H_17_N_3_O, the oxazine ring has a half-chair conformation. The dihedral angles between the best least-squares plane through the pyridine rings and the planar part (O–C–C–C–N) of the oxazine ring are 72.14 (6) and 35.44 (7)°, the smaller angle involving the pyridine ring adjacent to the oxazine O atom. The mol­ecule has two stereogenic centers at the oxazine carbons, *RS* and *SR*. The crystal packing reveals that symmetry-related mol­ecules are linked by inter­molecular N—H⋯N hydrogen bonds to form chains parallel to the *b* axis.

## Related literature

For related literature, see: Kurz *et al.* (2005 ▶); Turgut *et al.* (2007 ▶); Szatmari *et al.* (2003 ▶, 2004 ▶); Bernstein *et al.* (1995 ▶); Cremer & Pople (1975 ▶).
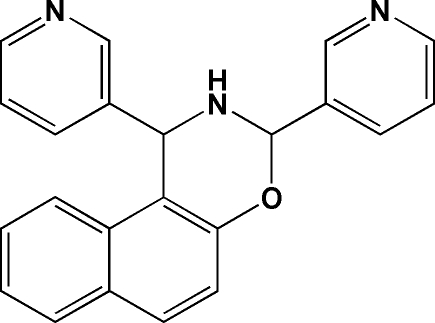


## Experimental

### 

#### Crystal data

C_22_H_17_N_3_O
*M* *_r_* = 339.39
Monoclinic, 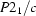

*a* = 12.1720 (8) Å
*b* = 8.0444 (6) Å
*c* = 18.7716 (15) Å
β = 112.615 (5)°
*V* = 1696.7 (2) Å^3^
*Z* = 4
Mo *K*α radiation
μ = 0.08 mm^−1^
*T* = 293 (2) K
0.28 × 0.22 × 0.12 mm

#### Data collection

Stoe IPDSII diffractometer
Absorption correction: none
15207 measured reflections
3703 independent reflections
1786 reflections with *I* > 2σ(*I*)
*R*_int_ = 0.124

#### Refinement

*R*[*F* ^2^ > 2σ(*F* ^2^)] = 0.041
*wR*(*F* ^2^) = 0.092
*S* = 0.80
3703 reflections
304 parameters
All H-atom parameters refined
Δρ_max_ = 0.12 e Å^−3^
Δρ_min_ = −0.13 e Å^−3^

### 

Data collection: *X-AREA* (Stoe & Cie, 2002 ▶); cell refinement: *X-AREA*; data reduction: *X-RED32* (Stoe & Cie, 2002 ▶); program(s) used to solve structure: *SHELXS97* (Sheldrick, 2008 ▶); program(s) used to refine structure: *SHELXL97* (Sheldrick, 2008 ▶); molecular graphics: *ORTEPIII* (Burnett & Johnson, 1996 ▶) and *PLATON* (Spek, 2003 ▶); software used to prepare material for publication: *WinGX* (Farrugia, 1999 ▶).

## Supplementary Material

Crystal structure: contains datablocks I, global. DOI: 10.1107/S1600536808003887/su2043sup1.cif

Structure factors: contains datablocks I. DOI: 10.1107/S1600536808003887/su2043Isup2.hkl

Additional supplementary materials:  crystallographic information; 3D view; checkCIF report

## Figures and Tables

**Table 1:** Hydrogen-bond geometry (Å, °)

*D*—H⋯*A*	*D*—H	H⋯*A*	*D*⋯*A*	*D*—H⋯*A*
N1—H1⋯N3^i^	0.98 (2)	2.04 (2)	3.009 (2)	170.7 (17)

Symmetry code: (i) 
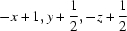
.

**Fig. 1.**
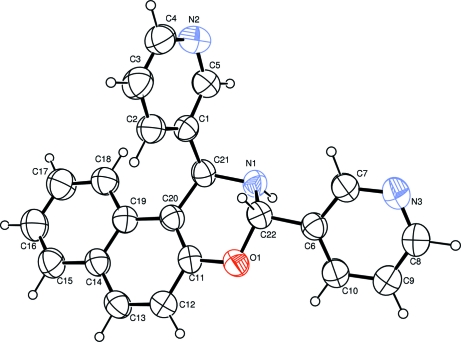
An ORTEPIII (Burnett & Johnson, 1996) view of the title compound with the atomic numbering scheme. Displacement ellipsoids are drawn at the 50% probability level. H atoms are shown as spheres of arbitrary size.

**Fig. 2.**
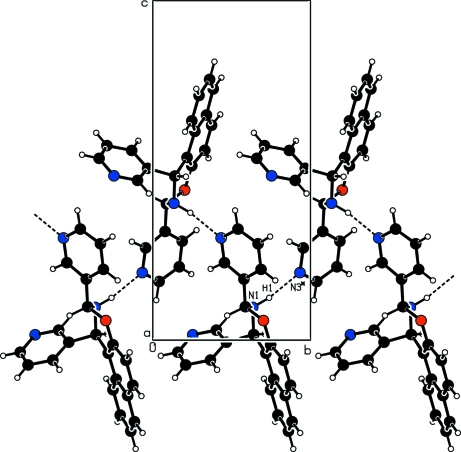
The crystal packing diagram of the title compound showing the infinite zigzag chain along the b axis, formed through N—H···N hydrogen bonding (dashed lines) [Symmetry codes: (*) 1 - x,1/2 + y,1/2 - z].

**Fig. 3.**
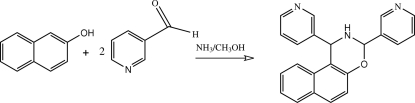
The formation of the title compound.

## supplementary crystallographic information

### Comment

1,3-oxazine heterocycles are of interest because they constitute an important class of natural and non-natural products. Many of them exhibit biological activity such as analgesic, anticonvulsant, antitubercular, antibacterial and anticancer (Kurz *et al.*, 2005; Turgut *et al.*, 2007). In addition, they can be used as intermediates in the synthesis of N-substituted amino alcohols or in enantioselective synthesis of chiral amines. The tautomeric character of the 1,3-O,*N*-heterocycles offers a great number of synthetic possibilities (Szatmari *et al.*, 2003; Szatmari *et al.*, 2004).

Atoms (C11, C20, C21 and O1) of the oxazine six-membered ring are planar to within 0.014 Å. The oxazine ring adopts a half-chair conformation, with atom C22 and N1 deviating by -0.375 (3) Å and 0.308 (3) Å, respectively, from the mean plane formed by atoms (C11, C20, C21 and O1). The ring puckering parameters for the oxazine ring are *Q* = 0.451 (2) Å, θ = 126.2 (2)° and φ = 92.4 (3) ° (Cremer & Pople, 1975). The dihedral angles made by the best least-squares plane through all six atoms of the oxazine ring with the mean planes of the pyridine rings (N2/C1—C5) and (N3/C6—C10) are 79.62 (9)° and 36.40 (9)°, respectively. The sum of the angles at N1 of the oxazine ring is 334.1°, in accordance with *sp*^3^-hybridization. The H atoms bonded to atoms C22 and C21 of the oxazine ring are axial and *trans* to one another. The structure is centrosymmetric so the absolute configurations of the two stereogenic centres, C21 and C22, are *RS* and S*R*, respectively.

In the crystal structure, intermolecular N1—H1···N3^i^ hydrogen bonds (Table 1) link the molecules to form an infinite one-dimensional polymeric chain. In this manner a C(6) chain (Bernstein *et al.*, 1995) is formed and the axis of the polymeric chain runs co-linear with the crystallographic [010] direction of the monoclinic unit cell (Fig. 2).

### Experimental

The title compound was prepared by cyclization reactions realised using 2-naphthol and pyridine-3-carbaldehyde in the presence of dry methanolic ammonia (Fig. 3). The structure of the title compound has been clarified by FTIR, MS and NMR techniques and confirmed by elemental analysis.

Pyridin-3-carbaldehyde (2 mmol; freshly distilled if liquid) and 25% methanolic ammonia solution (0.5 mL) were added to a solution of 2-naphthol (1 mmol) in absolute MeOH (0.5 ml). The mixture was left to stand at ambient temperature for 2 days, during which time a crystalline product separated out. The crude product was filtered off, washed with cold MeOH (2x2mL), then purified by column chromatography with ethyl acetate/n-hexane (3:1). Pale-yellow crystals of the title compound, suitable for X-ray analysis, were obtained by slow evaporation of this solution.

^1^H NMR (CDCl_3_, δ (p.p.m.)): 5.34 (s, 1H, CH); 5.89 (s, 1H, CH); 7.21–7.89 (m, 10H, ArH); 8.50–8.89 (m, 4H, pyridine)

^13^C NMR (CDCl_3_, δ (p.p.m.)): 56.23, 81.75, 124.37, 133.67, 139.76, 148.27, 150.52, 154.3

FTIR(KBr, cm^-1^, ν) 3335, 3056,3034, 1622,1596,1260,1233,1023,928

### Refinement

All H atoms were located in difference Fourier maps and were freely refined: N—H = 0.97 (2), C—H = 0.95 (2) - 1.04 (2) Å

### Crystal data

**Table:**

C_22_H_17_N_3_O	*F*_000_ = 712
*M**_r_* = 339.39	*D*_x_ = 1.329 Mg m^−^^3^
Monoclinic, *P*2_1_/*c*	Mo *K*α radiation λ = 0.71073 Å
Hall symbol: -P 2ybc	Cell parameters from 10289 reflections
*a* = 12.1720 (8) Å	θ = 1.7–27.2º
*b* = 8.0444 (6) Å	µ = 0.08 mm^−^^1^
*c* = 18.7716 (15) Å	*T* = 293 (2) K
β = 112.615 (5)º	Prism, pale yellow
*V* = 1696.7 (2) Å^3^	0.28 × 0.22 × 0.12 mm
*Z* = 4	

### Data collection

**Table:**

Stoe IPDSII diffractometer	3703 independent reflections
Radiation source: fine-focus sealed tube	1786 reflections with *I* > 2σ(*I*)
Monochromator: graphite	*R*_int_ = 0.124
Detector resolution: 6.67 pixels mm^-1^	θ_max_ = 27.2º
*T* = 293(2) K	θ_min_ = 2.4º
ω and φ scans	*h* = −15→15
Absorption correction: none	*k* = −10→10
15207 measured reflections	*l* = −24→24

### Refinement

**Table:**

Refinement on *F*^2^	Hydrogen site location: inferred from neighbouring sites
Least-squares matrix: full	All H-atom parameters refined
*R*[*F*^2^ > 2σ(*F*^2^)] = 0.041	*w* = 1/[σ^2^(*F*_o_^2^) + (0.0317*P*)^2^] where *P* = (*F*_o_^2^ + 2*F*_c_^2^)/3
*wR*(*F*^2^) = 0.092	(Δ/σ)_max_ < 0.001
*S* = 0.80	Δρ_max_ = 0.12 e Å^−^^3^
3703 reflections	Δρ_min_ = −0.13 e Å^−^^3^
304 parameters	Extinction correction: SHELXL97 (Sheldrick, 2008), Fc^*^=kFc[1+0.001xFc^2^λ^3^/sin(2θ)]^-1/4^
Primary atom site location: structure-invariant direct methods	Extinction coefficient: 0.0155 (15)
Secondary atom site location: difference Fourier map	

### Special details

**Table:**

Geometry. All e.s.d.'s (except the e.s.d. in the dihedral angle between two l.s. planes) are estimated using the full covariance matrix. The cell e.s.d.'s are taken into account individually in the estimation of e.s.d.'s in distances, angles and torsion angles; correlations between e.s.d.'s in cell parameters are only used when they are defined by crystal symmetry. An approximate (isotropic) treatment of cell e.s.d.'s is used for estimating e.s.d.'s involving l.s. planes.
Refinement. Refinement of *F*^2^ against ALL reflections. The weighted *R*-factor *wR* and goodness of fit *S* are based on *F*^2^, conventional *R*-factors *R* are based on *F*, with *F* set to zero for negative *F*^2^. The threshold expression of *F*^2^ > σ(*F*^2^) is used only for calculating *R*-factors(gt) *etc*. and is not relevant to the choice of reflections for refinement. *R*-factors based on *F*^2^ are statistically about twice as large as those based on *F*, and *R*- factors based on ALL data will be even larger.

### Fractional atomic coordinates and isotropic or equivalent isotropic displacement parameters (Å^2^)

**Table:**

	*x*	*y*	*z*	*U*_iso_*/*U*_eq_	
O1	0.50525 (11)	0.70176 (16)	0.05782 (6)	0.0613 (3)	
N1	0.34476 (13)	0.63357 (19)	0.09666 (7)	0.0530 (4)	
N2	0.07931 (16)	0.2535 (2)	0.01583 (9)	0.0797 (5)	
N3	0.61988 (16)	0.4333 (2)	0.30101 (8)	0.0708 (5)	
C1	0.20165 (16)	0.4668 (2)	−0.00607 (8)	0.0515 (4)	
C2	0.22195 (18)	0.3658 (2)	−0.05879 (10)	0.0624 (5)	
C3	0.1718 (2)	0.2087 (3)	−0.07310 (11)	0.0721 (6)	
C4	0.1012 (2)	0.1592 (3)	−0.03549 (11)	0.0750 (6)	
C5	0.13038 (19)	0.4030 (3)	0.02947 (10)	0.0664 (5)	
C6	0.55109 (16)	0.5779 (2)	0.17981 (8)	0.0518 (4)	
C7	0.54203 (19)	0.4545 (2)	0.22813 (10)	0.0619 (5)	
C8	0.7097 (2)	0.5402 (3)	0.32714 (11)	0.0717 (6)	
C9	0.7258 (2)	0.6678 (3)	0.28396 (10)	0.0713 (6)	
C10	0.64504 (18)	0.6866 (2)	0.20842 (9)	0.0621 (5)	
C11	0.42404 (16)	0.7472 (2)	−0.01352 (8)	0.0521 (4)	
C12	0.4760 (2)	0.8298 (2)	−0.05937 (10)	0.0581 (5)	
C13	0.40609 (19)	0.8731 (2)	−0.13285 (10)	0.0614 (5)	
C14	0.28339 (18)	0.8404 (2)	−0.16314 (9)	0.0571 (5)	
C15	0.2110 (2)	0.8834 (3)	−0.24001 (10)	0.0703 (6)	
C16	0.0929 (2)	0.8602 (3)	−0.26809 (12)	0.0854 (7)	
C17	0.0389 (2)	0.7933 (3)	−0.22063 (12)	0.0884 (7)	
C18	0.10616 (19)	0.7473 (3)	−0.14611 (10)	0.0703 (6)	
C19	0.23078 (17)	0.7670 (2)	−0.11493 (9)	0.0548 (5)	
C20	0.30495 (16)	0.7173 (2)	−0.03826 (8)	0.0506 (4)	
C21	0.25461 (17)	0.6395 (2)	0.01656 (8)	0.0530 (4)	
C22	0.45819 (16)	0.5869 (2)	0.09848 (8)	0.0526 (4)	
H1	0.3522 (17)	0.738 (3)	0.1247 (10)	0.080 (6)*	
H2	0.2726 (17)	0.408 (2)	−0.0873 (9)	0.073 (5)*	
H3	0.1834 (19)	0.141 (3)	−0.1108 (12)	0.099 (7)*	
H4	0.0621 (19)	0.051 (3)	−0.0461 (11)	0.089 (7)*	
H5	0.1146 (18)	0.475 (3)	0.0680 (10)	0.083 (6)*	
H7	0.4767 (18)	0.367 (3)	0.2099 (10)	0.077 (6)*	
H8	0.7714 (19)	0.522 (3)	0.3822 (11)	0.094 (7)*	
H9	0.7932 (19)	0.744 (3)	0.3052 (10)	0.083 (6)*	
H10	0.6560 (18)	0.777 (3)	0.1759 (10)	0.090 (6)*	
H12	0.5600 (18)	0.853 (2)	−0.0360 (9)	0.064 (5)*	
H13	0.4472 (17)	0.928 (2)	−0.1648 (9)	0.076 (5)*	
H15	0.2541 (18)	0.935 (3)	−0.2732 (10)	0.087 (6)*	
H16	0.041 (2)	0.891 (3)	−0.3203 (13)	0.113 (8)*	
H17	−0.047 (2)	0.776 (3)	−0.2404 (12)	0.107 (8)*	
H18	0.0647 (16)	0.705 (2)	−0.1138 (9)	0.066 (5)*	
H21	0.1904 (15)	0.710 (2)	0.0184 (8)	0.055 (5)*	
H22	0.4510 (15)	0.472 (2)	0.0725 (8)	0.057 (5)*	

### Atomic displacement parameters (Å^2^)

**Table:**

	*U*^11^	*U*^22^	*U*^33^	*U*^12^	*U*^13^	*U*^23^
O1	0.0548 (8)	0.0777 (9)	0.0510 (6)	−0.0024 (6)	0.0199 (6)	0.0111 (6)
N1	0.0553 (10)	0.0593 (10)	0.0451 (7)	−0.0015 (7)	0.0201 (7)	−0.0059 (6)
N2	0.0853 (14)	0.0791 (13)	0.0802 (10)	−0.0210 (10)	0.0380 (10)	−0.0002 (9)
N3	0.0791 (13)	0.0742 (12)	0.0544 (9)	−0.0034 (10)	0.0205 (8)	0.0110 (7)
C1	0.0464 (11)	0.0572 (11)	0.0479 (8)	−0.0006 (8)	0.0147 (8)	0.0004 (7)
C2	0.0677 (14)	0.0614 (12)	0.0625 (10)	−0.0053 (10)	0.0296 (10)	−0.0082 (9)
C3	0.0791 (16)	0.0637 (13)	0.0711 (12)	−0.0062 (11)	0.0261 (11)	−0.0124 (10)
C4	0.0768 (16)	0.0669 (14)	0.0712 (12)	−0.0135 (12)	0.0172 (11)	0.0009 (11)
C5	0.0717 (15)	0.0705 (13)	0.0615 (10)	−0.0078 (11)	0.0305 (10)	−0.0007 (10)
C6	0.0556 (12)	0.0525 (10)	0.0492 (9)	0.0008 (9)	0.0223 (8)	−0.0024 (7)
C7	0.0701 (15)	0.0624 (12)	0.0539 (10)	−0.0053 (10)	0.0246 (10)	0.0031 (9)
C8	0.0690 (16)	0.0815 (15)	0.0561 (11)	−0.0004 (12)	0.0146 (10)	0.0068 (10)
C9	0.0666 (15)	0.0794 (15)	0.0596 (11)	−0.0132 (12)	0.0152 (10)	0.0003 (10)
C10	0.0683 (14)	0.0639 (12)	0.0529 (10)	−0.0049 (10)	0.0217 (9)	0.0019 (9)
C11	0.0582 (13)	0.0539 (10)	0.0433 (8)	0.0026 (9)	0.0183 (8)	0.0005 (7)
C12	0.0592 (14)	0.0595 (11)	0.0568 (10)	−0.0026 (10)	0.0236 (9)	0.0017 (8)
C13	0.0733 (15)	0.0580 (11)	0.0573 (10)	−0.0009 (10)	0.0301 (10)	0.0073 (8)
C14	0.0625 (14)	0.0563 (11)	0.0516 (9)	0.0025 (9)	0.0209 (9)	0.0041 (8)
C15	0.0777 (18)	0.0720 (13)	0.0576 (11)	0.0021 (11)	0.0219 (11)	0.0106 (9)
C16	0.0798 (19)	0.1046 (18)	0.0596 (12)	−0.0013 (14)	0.0131 (12)	0.0173 (12)
C17	0.0630 (18)	0.119 (2)	0.0697 (13)	0.0006 (14)	0.0104 (11)	0.0228 (12)
C18	0.0620 (15)	0.0829 (14)	0.0636 (11)	−0.0025 (11)	0.0214 (10)	0.0088 (10)
C19	0.0585 (14)	0.0518 (10)	0.0526 (9)	0.0029 (9)	0.0196 (8)	0.0003 (8)
C20	0.0554 (13)	0.0495 (10)	0.0484 (8)	0.0016 (8)	0.0215 (8)	−0.0003 (7)
C21	0.0542 (12)	0.0591 (11)	0.0475 (9)	0.0053 (9)	0.0217 (8)	0.0007 (8)
C22	0.0566 (13)	0.0551 (11)	0.0480 (9)	0.0021 (9)	0.0222 (8)	0.0009 (8)

### Geometric parameters (Å, °)

**Table:**

O1—C11	1.3732 (19)	C9—C10	1.389 (3)
O1—C22	1.4492 (19)	C9—H9	0.98 (2)
N1—C22	1.419 (2)	C10—H10	0.99 (2)
N1—C21	1.483 (2)	C11—C20	1.363 (2)
N1—H1	0.97 (2)	C11—C12	1.415 (2)
N2—C4	1.331 (3)	C12—C13	1.359 (3)
N2—C5	1.332 (2)	C12—H12	0.962 (19)
N3—C8	1.328 (3)	C13—C14	1.404 (3)
N3—C7	1.342 (2)	C13—H13	1.017 (18)
C1—C2	1.374 (2)	C14—C15	1.414 (2)
C1—C5	1.381 (2)	C14—C19	1.422 (2)
C1—C21	1.522 (2)	C15—C16	1.341 (3)
C2—C3	1.384 (3)	C15—H15	1.040 (19)
C2—H2	1.017 (17)	C16—C17	1.402 (3)
C3—C4	1.365 (3)	C16—H16	0.97 (2)
C3—H3	0.95 (2)	C17—C18	1.372 (3)
C4—H4	0.98 (2)	C17—H17	0.98 (2)
C5—H5	1.002 (19)	C18—C19	1.409 (3)
C6—C10	1.374 (2)	C18—H18	0.988 (17)
C6—C7	1.378 (2)	C19—C20	1.431 (2)
C6—C22	1.512 (2)	C20—C21	1.520 (2)
C7—H7	1.02 (2)	C21—H21	0.975 (16)
C8—C9	1.368 (3)	C22—H22	1.032 (17)
C8—H8	1.03 (2)		
			
C11—O1—C22	113.65 (13)	C13—C12—C11	119.1 (2)
C22—N1—C21	111.52 (12)	C13—C12—H12	123.7 (10)
C22—N1—H1	109.1 (11)	C11—C12—H12	117.2 (10)
C21—N1—H1	113.5 (11)	C12—C13—C14	121.13 (17)
C4—N2—C5	116.46 (18)	C12—C13—H13	116.8 (11)
C8—N3—C7	116.90 (17)	C14—C13—H13	122.0 (10)
C2—C1—C5	116.69 (17)	C13—C14—C15	121.03 (17)
C2—C1—C21	124.50 (16)	C13—C14—C19	119.30 (15)
C5—C1—C21	118.79 (15)	C15—C14—C19	119.65 (19)
C1—C2—C3	119.44 (18)	C16—C15—C14	121.3 (2)
C1—C2—H2	119.6 (10)	C16—C15—H15	122.1 (11)
C3—C2—H2	120.9 (10)	C14—C15—H15	116.6 (11)
C4—C3—C2	119.0 (2)	C15—C16—C17	119.8 (2)
C4—C3—H3	121.6 (14)	C15—C16—H16	123.2 (14)
C2—C3—H3	119.3 (13)	C17—C16—H16	116.9 (14)
N2—C4—C3	123.3 (2)	C18—C17—C16	120.6 (2)
N2—C4—H4	116.1 (12)	C18—C17—H17	118.8 (13)
C3—C4—H4	120.6 (12)	C16—C17—H17	120.5 (12)
N2—C5—C1	125.15 (18)	C17—C18—C19	121.2 (2)
N2—C5—H5	117.1 (11)	C17—C18—H18	118.3 (10)
C1—C5—H5	117.7 (11)	C19—C18—H18	120.5 (10)
C10—C6—C7	117.81 (16)	C18—C19—C14	117.29 (16)
C10—C6—C22	123.44 (15)	C18—C19—C20	123.26 (16)
C7—C6—C22	118.75 (17)	C14—C19—C20	119.45 (17)
N3—C7—C6	124.0 (2)	C11—C20—C19	118.07 (14)
N3—C7—H7	114.2 (10)	C11—C20—C21	119.66 (14)
C6—C7—H7	121.8 (10)	C19—C20—C21	122.23 (17)
N3—C8—C9	123.56 (19)	N1—C21—C20	111.47 (15)
N3—C8—H8	117.4 (12)	N1—C21—C1	108.81 (14)
C9—C8—H8	119.0 (12)	C20—C21—C1	115.12 (13)
C8—C9—C10	118.7 (2)	N1—C21—H21	105.9 (9)
C8—C9—H9	121.3 (11)	C20—C21—H21	108.8 (9)
C10—C9—H9	120.0 (11)	C1—C21—H21	106.2 (10)
C6—C10—C9	119.09 (18)	N1—C22—O1	113.38 (14)
C6—C10—H10	121.0 (11)	N1—C22—C6	112.33 (12)
C9—C10—H10	119.9 (12)	O1—C22—C6	105.59 (14)
C20—C11—O1	123.91 (14)	N1—C22—H22	108.7 (9)
C20—C11—C12	122.79 (15)	O1—C22—H22	107.5 (8)
O1—C11—C12	113.30 (17)	C6—C22—H22	109.2 (9)
			
C5—C1—C2—C3	−0.3 (3)	C13—C14—C19—C18	175.59 (17)
C21—C1—C2—C3	178.03 (18)	C15—C14—C19—C18	−2.6 (3)
C1—C2—C3—C4	1.4 (3)	C13—C14—C19—C20	−4.4 (2)
C5—N2—C4—C3	0.1 (3)	C15—C14—C19—C20	177.40 (16)
C2—C3—C4—N2	−1.3 (3)	O1—C11—C20—C19	−178.17 (14)
C4—N2—C5—C1	1.0 (3)	C12—C11—C20—C19	2.2 (2)
C2—C1—C5—N2	−0.9 (3)	O1—C11—C20—C21	4.2 (2)
C21—C1—C5—N2	−179.35 (18)	C12—C11—C20—C21	−175.39 (15)
C8—N3—C7—C6	0.7 (3)	C18—C19—C20—C11	−178.01 (17)
C10—C6—C7—N3	−0.6 (3)	C14—C19—C20—C11	1.9 (2)
C22—C6—C7—N3	179.19 (17)	C18—C19—C20—C21	−0.5 (3)
C7—N3—C8—C9	−0.1 (3)	C14—C19—C20—C21	179.47 (16)
N3—C8—C9—C10	−0.6 (3)	C22—N1—C21—C20	−41.2 (2)
C7—C6—C10—C9	−0.1 (3)	C22—N1—C21—C1	86.79 (17)
C22—C6—C10—C9	−179.90 (17)	C11—C20—C21—N1	10.0 (2)
C8—C9—C10—C6	0.7 (3)	C19—C20—C21—N1	−167.51 (15)
C22—O1—C11—C20	13.0 (2)	C11—C20—C21—C1	−114.56 (18)
C22—O1—C11—C12	−167.37 (14)	C19—C20—C21—C1	67.9 (2)
C20—C11—C12—C13	−3.9 (3)	C2—C1—C21—N1	−109.53 (19)
O1—C11—C12—C13	176.41 (15)	C5—C1—C21—N1	68.8 (2)
C11—C12—C13—C14	1.3 (3)	C2—C1—C21—C20	16.4 (3)
C12—C13—C14—C15	−179.09 (18)	C5—C1—C21—C20	−165.26 (17)
C12—C13—C14—C19	2.7 (3)	C21—N1—C22—O1	61.15 (19)
C13—C14—C15—C16	−176.6 (2)	C21—N1—C22—C6	−179.25 (14)
C19—C14—C15—C16	1.6 (3)	C11—O1—C22—N1	−46.19 (18)
C14—C15—C16—C17	0.5 (4)	C11—O1—C22—C6	−169.58 (13)
C15—C16—C17—C18	−1.6 (4)	C10—C6—C22—N1	−111.91 (19)
C16—C17—C18—C19	0.5 (4)	C7—C6—C22—N1	68.3 (2)
C17—C18—C19—C14	1.6 (3)	C10—C6—C22—O1	12.1 (2)
C17—C18—C19—C20	−178.5 (2)	C7—C6—C22—O1	−167.64 (15)

### Hydrogen-bond geometry (Å, °)

**Table:**

*D*—H···*A*	*D*—H	H···*A*	*D*···*A*	*D*—H···*A*
N1—H1···N3^i^	0.98 (2)	2.04 (2)	3.009 (2)	170.7 (17)

Symmetry codes: (i) −*x*+1, *y*+1/2, −*z*+1/2.
